# Reduced resting state functional connectivity with increasing age-related hearing loss and McGurk susceptibility

**DOI:** 10.1038/s41598-020-74012-0

**Published:** 2020-10-12

**Authors:** Alina Schulte, Christiane M. Thiel, Anja Gieseler, Maike Tahden, Hans Colonius, Stephanie Rosemann

**Affiliations:** 1 Biological Psychology, Department of Psychology, School of Medicine and Health Sciences, Carl-Von-Ossietzky Universität Oldenburg, Ammerländer Heerstraße 114-118, 26111 Oldenburg, Germany; 2 Cluster of Excellence “Hearing4all”, Carl Von Ossietzky Universität Oldenburg, Oldenburg, Germany; 3 Cognitive Psychology, Department of Psychology, School of Medicine and Health Sciences, Carl-Von-Ossietzky Universität Oldenburg, Oldenburg, Germany

**Keywords:** Neuroscience, Psychology

## Abstract

Age-related hearing loss has been related to a compensatory increase in audio-visual integration and neural reorganization including alterations in functional resting state connectivity. How these two changes are linked in elderly listeners is unclear. The current study explored modulatory effects of hearing thresholds and audio-visual integration on resting state functional connectivity. We analysed a large set of resting state data of 65 elderly participants with a widely varying degree of untreated hearing loss. Audio-visual integration, as gauged with the McGurk effect, increased with progressing hearing thresholds. On the neural level, McGurk illusions were negatively related to functional coupling between motor and auditory regions. Similarly, connectivity of the dorsal attention network to sensorimotor and primary motor cortices was reduced with increasing hearing loss. The same effect was obtained for connectivity between the salience network and visual cortex. Our findings suggest that with progressing untreated age-related hearing loss, functional coupling at rest declines, affecting connectivity of brain networks and areas associated with attentional, visual, sensorimotor and motor processes. Especially connectivity reductions between auditory and motor areas were related to stronger audio-visual integration found with increasing hearing loss.

## Introduction

Sensory impairments among elderly are a substantial health issue with increasing relevance in an ageing society. With respect to hearing difficulties, a prevalence of 75% has been described for hearing loss in the US population above an age of 70^1^. Age-related hearing loss, also known as presbycusis, is a form of bilateral sensorineural hearing loss typically affecting high frequencies from 2000 Hz onwards^2^. The degraded auditory input in that frequency range leads to difficulties in speech perception and makes conversations highly demanding, requiring additional attentional and cognitive resources^3,4^. Age-related hearing loss is also associated with an accelerated loss of cognitive abilities and a higher risk of dementia^5–8^ and may further result in depressive symptoms and social isolation^9^. Together with behavioural symptoms, changes in brain structure and neural activity patterns have been described in presbycusis patients. These involve decreased neural activity in temporal brain regions together with increased frontal activation in response to auditory stimuli^10^, as well as atrophy^11^ and cross-modal plasticity^12^ of auditory cortices. Moreover, functional coupling of several areas and networks are altered in participants with age-related hearing loss^13,14^. Respective investigations of functional coupling at rest are, however, scarce and revealed inconsistent results, which the present study aims to disentangle studying a large sample of elderly normal and hard of hearing participants who were not using hearing aids yet.

Resting state functional connectivity measures temporal correlations of spontaneous, low-frequency neuronal activity between anatomically separated brain areas in absence of any task^15–17^. This way, brain regions are organized in several functional units such as the salience network, the default mode or the dorsal attention network. The salience network encompasses nodes in the cingulate cortex and the insula as well as prefrontal and supramarginal regions and is especially involved in the detection of salient events^18^. In elderly participants, weakened salience network connectivity has been related to cognitive decline and ageing^19^ and is possibly also influenced by hearing impairment^4^. For several other networks, connectivity modifications have been reported in hard-of-hearing individuals^14,16,20,21^. Specifically for age-related hearing loss, differences in network connectivity have been reported for the default mode network (DMN) and the dorsal attention network (DAN). The DMN is associated with internal thoughts and daydreaming as it is highly active during rest^22,23^. It includes prefrontal, temporal-parietal, and cingulate nodes^24,25^. In contrast, regions of the DAN, encompassing parts of the frontal eye fields and intraparietal sulci^26^, exhibit, like those of the salience network, reduced activity during rest, but elevated activity in attention-requiring tasks. The DAN is specifically recruited during demanding cognitive tasks as well as in guiding selective attention and stimulus responses^27^. Previous findings comprise increased coupling of the DMN to the middle frontal gyrus, but reduced connectivity of the DAN to the insula and postcentral gyrus in hard-of-hearing elderly^16^. Chen and colleagues^14^ reported a negative correlation of connectivity within the superior temporal gyrus and hearing thresholds. Findings from Rosemann and Thiel^13^ suggested that listening effort, rather than hearing thresholds, might be responsible for alterations of resting state connectivity in hard-of-hearing elderly. Reduced coupling of the DAN to the precuneus and superior parietal lobule as well as the auditory cortices to the inferior frontal gyrus were associated with subjective listening effort. In another study^21^, connectivity between auditory and visual areas (MT+) was positively related to hearing thresholds during both resting state and audio-visual processing. In summary, the literature provides evidence for changes in brain connectivity between several areas and networks associated with hearing loss. Existing results do, however, not yield a coherent picture which may be due to the small sample size of prior studies. Details about the directionality of the effect and its relation to behavioural changes remain unclear.

Besides neural changes, audio-visual integration abilities are more pronounced among hard-of-hearing individuals compared to normal-hearing populations^28,30^. In line with the principle of inverse effectiveness^31^ the lacking accuracy in auditory perception and therefore increased ambiguity of stimuli leads to enhanced multisensory integration. Accordingly, particularly for older adults with decreasing hearing abilities, additional visual speech information conveyed via lip and mouth movements gains importance in order to increase speech intelligibility^32–34^. Integration can also occur for incongruent auditory and visual information. The McGurk effect^35,36^ constitutes a powerful example of this phenomenon. When presented with the visual image of a speaker producing lip movements of a syllable together with a different syllable presented for the auditory modality, listeners often perceive a sound that corresponds to a fused percept of both. For instance, if a 'ba' sound is added on a video sequence showing lip movements of someone producing a 'ga' sound, many people report to hear a 'da' sound^37^.

Early auditory and visual areas are involved in the generation of the fused McGurk percept in healthy participants^38^. Additionally, areas crucial for speech production, including the lip area of the primary motor cortex, have been found to be involved in the perception of audio-visual speech stimuli^39–42^. Thereby, bottom-up sensory input is believed to be integrated with top-down information from frontal and motor areas in the posterior superior temporal sulcus (STS)^38,43,44^. Functional connectivity of the STS to sensory cortices was increased and depended on whether the reliability of the auditory or the visual stimulus part was less noisy and accordingly more reliable in audio-visual speech stimuli. However, when specifically considering McGurk susceptibility, connectivity between the STS and frontal, auditory or visual cortices was not modulated by illusion rates^45^. With regard to the McGurk illusion in hard-of-hearing subjects, electrophysiological evidence of cross-modal activity in auditory areas in response to the visual face stimulus of the paradigm was demonstrated^30^. Furthermore, audio-visual integration in hard-of-hearing participants has been linked to elevated activity in frontal brain regions and the left postcentral gyrus^28^ as well as task-based connectivity alterations of the auditory cortex to middle frontal, occipital and supramarginal gyri^29^. However, whether these connectivity modulations are persistent at rest and to what extent alterations in cross-modal activity patterns are mirrored in functional resting state connectivity remains unknown. In order to advance hearing aid fitting options, understanding neural adaptations of already mild stages of age-related hearing loss is of crucial relevance.

To date, only a very limited number of studies investigated resting state connectivity in age-related hearing loss and research on the McGurk effect in this context is entirely lacking. The present research explores, for the first time, modulatory effects of hearing thresholds and McGurk susceptibility on resting state connectivity in a larger sample (n = 65) of elderly participants and thus aims to disentangle previous ambiguous findings. Based on existing literature, we hypothesised that audio-visual integration abilities correlate positively with high-frequency hearing loss^28,30^. Concerning the neuroimaging data, we expected variability in resting state connectivity of the auditory cortices^13,21^ as well as the DAN^13,20^, DMN^13^, and salience network^13,19^ to be explained by progressing hearing loss. Further, we assumed that resting state functional coupling of audio-visual integration related areas, such as the primary motor cortex, STS, and auditory cortices, changes as a function of McGurk susceptibility^28,41,45^.

## Methods

### Participants

Combining two samples from previous studies, data from *n* = 65 elderly participants (30 male, 35 female) were analysed. Overall, participants’ age ranged from 52 to 80 years with a mean age of 66.82 years (*SD* = 5.89). Data of 36 subjects was recorded by Rosemann and Thiel^13,28^ and included 19 normal-hearing participants and 17 subjects with a mild-to-moderate hearing loss. Data of the remaining 29 mild-to-severe hearing-impaired subjects were measured within the project "Hearing aid use, audiovisual integration, and speech performance: probing the interplay", funded by the Hearing Industry Research Consortium (IRC) Grant 2017. Participants with a high-frequency hearing loss of more than 30 dB HL for the frequencies between 2000 and 8000 Hz were considered to have presbycusis (cf. WHO, 2001 definition of hearing loss^47^). Hearing thresholds for the lower frequencies (125–1000 Hz) were within a normal range for all subjects and only subjects with a symmetrical hearing deprivation (less than 10 dB HL difference between both ears) were included in the analysis. All subjects were right-handed, German native speakers and reported no current or previous use of a hearing aid. Further eligibility criteria required individuals to have no current or previous neurological or psychiatric disorders (self-report) and no contraindications for the MRI, for instance pacemakers or stents. Both studies were approved by the local ethics committee of the University of Oldenburg and conducted in accordance with the Declaration of Helsinki 2008**.** All participants gave written and informed consent and were paid for their participation.

### Experimental procedure

Both studies consisted of MRI measurements as well as neuropsychological assessments and the audiometric testing. In the subsample provided by Rosemann and Thiel^13,28^, resting state and anatomical MRI was recorded after a task-based MRI session and was followed by questionnaires. Afterwards, further behavioural measures including the McGurk task and pure-tone audiometry were assessed.

In the IRC project, measurements took place on two independent testing days within 1 week. Amongst other assessments, the McGurk task and audiometry were conducted on one day, whereas MRI recordings together with additional questionnaires were conducted on the second date.

In both projects, hearing thresholds were obtained using air conduction pure tone audiometry for the frequencies 125, 250, 500, 1000, 2000, 4000, 6000 and 8000 Hz.

### Stimuli

Stimulus material of the McGurk task was recorded in the Department of Media Production at the University of Oldenburg and contained audio and video files of a male speaker producing different syllables. The sound of the spoken syllable and visual information of the speaker’s face showing his articulatory lip movements were presented either in isolation (auditory-only and visual-only conditions), combined correctly (congruent conditions) or combined to incongruent audio-visual stimulus conditions (McGurk trials). Only incongruent trials were evaluated in order to determine McGurk illusion rates. The ratio of perceived illusions in incongruent trials served as an indicator for individual audio-visual integration abilities.

In both datasets, participants performed the McGurk task outside of the scanner using the same stimulus material, whereas the presented syllables and the timing of presentation differed slightly: The IRC study included auditory ‘ba’ sounds and visual ‘ga’ stimuli leading to the illusionary, fused percept of ‘da’. Participants were asked to indicate which sound they perceived in a three-alternative forced choice task including all three syllables as possible answers. In total, the experiment consisted of 200 trials: 90 incongruent and 90 congruent trials (45 for each syllable, respectively) and 10 auditory only and 10 visual only trials (5 for each syllable, respectively). The combined audio-visual stimuli were presented either simultaneously or with different Stimulus Onset Asynchronies (SOAs), in which the video sequence followed the auditory stimulus presentation. For both congruent and incongruent audio-visual stimuli, there were 10 trials with SOAs of 0, 70, 120, 170, 220, 270, 320, 370, 420 ms each. A previous analysis revealed that the effect of hard-of-hearing participants perceiving significantly more illusions than normal-hearing subjects is present across all SOAs and stronger for shorter SOAs (Gieseler et al., unpublished observations). Approaching the trade-off of including preferably trials without SOAs only (to assimilate both datasets best possible), while ensuring a sufficient number of trials, trials with SOAs of 0, 70 and 120 ms were analysed, resulting in 30 congruent and 30 incongruent audio-visual trials. Sound intensity of the stimuli was adjusted by each subject individually. The default intensity of 70 dB SPL could be modified by the participant in steps of 1 dB SPL to maximally 90 dB SPL. Stimuli were presented with 69.41 dB SPL on average, ranging from 65 dB SPL to 78 dB SPL.

In the study by Rosemann and Thiel^28^, in addition to the combination of auditory ’ba’ and visual ’ga’ syllables, auditory ’ba’ sounds were paired with visual ’ta’ stimuli, which could also lead to the perception of the phoneme ’da’. Moreover, an illusionary percept of ’ta’ was produced by using auditory ’pa’ and visual ’ka’ stimuli. Fifteen trials per syllable pair were presented resulting in 45 incongruent trials in total. No SOAs were used in this experiment. For all six syllables, there were seven congruent audio-visual trials, auditory-only and visual-only trials each, leading to 42 presentations for each condition and 171 trials in total. Answers were given in a four-alternative forced-choice task in which the illusionary syllable as well as the presented visual and auditory syllable were among the four response options for incongruent trials. Answers were selected using the numbers one to four on the keyboard with alternating response options across conditions and trials. Here, stimuli were presented at a constant level of 68 dB SPL for all subjects. Stimuli were presented using the Presentation software (Version 18.3, Neurobehavioral Systems, Inc., Berkeley, CA, www.neurobs.com).

### Data acquisition

Imaging data were acquired on a 3 T whole-body Siemens Magnetom Prisma MRI machine with a 20-channel head coil. Participants fixated a white dot, positioned centrally on a black screen, while resting state data was recorded with an ascending echo planar imaging sequence (320 T2*-weighted volumes, TR = 1500 ms, TE = 30 ms, voxel size = 2.2 × 2.2 × 3 mm, 25 slices). A 3D T1-weighted sequence with MP-RAGE was used for co-registration to structural images. Note that the sequences differed slightly in the two underlying datasets (TR = 2300/2000 ms, TE = 4.16/2.07, voxel size 1 × 1 × 1 mm/0.75 × 0.75 × 0.75 mm, 176/224 sagittal slices).

### Data analysis

To assess the relationship between hearing-impairment and audio-visual integration abilities, a correlation analysis was computed. Additionally, a partial correlation analysis of hearing thresholds and McGurk illusion rates whilst controlling for age effects was performed. Pearson’s correlation coefficients were tested two-tailed and determined to be significant when passing a threshold of α = 0.05. Statistical analyses of behavioural data were performed using IBM SPSS Statistics 24.

Resting state fMRI data were analysed with the Statistical Parametric Mapping software package (SPM12, Wellcome Department of Imaging Neuroscience, London, UK) based on MATLAB 2016b (MathWorks, Natick, MA, USA) and the CONN toolbox^46^ for SPM. Images were pre-processed in SPM including spatial realignment estimation, slice timing correction to the first slice of the volume and co-registration. These steps were followed by normalization to the Montreal Neurological Institute space using parameters obtained from segmentation of the anatomical T1-weighted image and spatial smoothing using a Gaussian kernel with a full width at half maximum of 8 mm. Next, data processing proceeded using the CONN toolbox. For a more thorough cleaning of the data, remaining physiological and movement artefacts were removed by linear regression. BOLD signal from white matter and cerebrospinal fluid as well as realignment parameters were used for denoising. Subsequently, a band-pass filter (0.008–0.9 Hz) and linear detrending was applied. First-level analyses revealed Fisher-transformed correlation coefficients for each subject. In second-level analyses, the individual seed-to-voxel connectivity maps of a specific seed to the whole brain were entered into regression models to test the hypotheses of changed resting state functional connectivity with hearing impairment or audio-visual integration abilities.

Relevant resting state networks included the DAN, DMN, and the salience network. Masks of these network seeds were provided by the atlas implemented in CONN (the FSL Harvard–Oxford atlas was used for cortical and subcortical areas). All nodes of a network were equally weighted, contributing jointly to the seed network's connectivity. Based on previous findings in the literature, additional seed areas were defined using the Automated Anatomical Labeling (AAL) ROI-Library^47^ within the WFU Pickatlas^48^. An auditory seed, defined by both left and right Brodmann areas 41 and 42, was created. Connectivity as a function of hearing loss was tested for the auditory cortices as well as the three aforementioned network seeds. For correlation analyses of brain connectivity with McGurk illusion rates, the same auditory seed was used as well as two other areas related to audio-visual integration, the left motor lip area and the left STS. The seed coordinates for the lip area were based on the average individual activation peaks in response to the McGurk task from Murakami and associates^41^. For the seed in the left STS, coordinates from the same study, used in an effective connectivity measure, were adopted. The masks of these two seed regions constitute spheres of 8 mm radius around the coordinates provided by the literature (ibid.). All seed regions and networks investigated are listed in Table 1. Regression analyses of connectivity were performed with hearing thresholds and McGurk illusion rates as the regressor and age as a covariate to control for age effects. Two-sided contrasts with a height threshold of *p* < 0.001 and a cluster forming threshold of *pFWE* < 0.05 were calculated. Further, a Bonferroni-correction was applied resulting in adjusted significance thresholds of *p* < 0.0125 (divided by the four seed regions used) for the models with hearing loss as a regressor. Resting state connectivity analyses with respect to the McGurk task were performed for three seed areas resulting in a corrected significance threshold of *p* < 0.016.

**Table 1:** Seed regions for the different networks used in the resting state analysis.

Network	Brain region	MNI coordinates
**Hearing loss**		
Default mode network	Medial prefrontal cortex	(1, 55, − 3)
	Parietal lobe (left)	(− 39, − 77, 33)
	Parietal lobe (right)	(47, − 67, 29)
	Posterior cingulate (right)	(1, − 61, 38)
Salience network	Anterior cingulate	(0, 22, 35)
	Anterior insula (left)	(− 44, 13, 1)
	Anterior insula (right)	(47, 14, 0)
	Prefrontal cortex (left)	(− 32, 45, 27)
	Prefrontal cortex (right)	(32, 46, 27)
	Supramarginal gyrus (left)	(− 60, − 39, 31)
	Supramarginal gyrus (right)	(62, − 35, 32)
Dorsal attention network	Frontal eye field (left)	(− 27, − 9, 64)
	Frontal eye field (right)	(30, − 6, 64)
	Intraparietal sulcus (left)	(− 39, − 43, 52)
	Intraparietal sulcus (right)	(39, − 42, 54)
Auditory cortices	Brodmann area 41 (left)	
	Brodmann area 41 (right)	
	Brodmann area 42 (left)	
	Brodmann area 42 (right)	
**McGurk**		
Auditory cortices	Brodmann area 41 (left)	
	Brodmann area 41 (right)	
	Brodmann area 42 (left)	
	Brodmann area 42 (right)	
Primary motor cortex	Lip area (left)	(− 44, − 11, 34)
Superior temporal sulcus	Left	(− 50, − 62, 18)

## Results

### Behavioural data

#### Hearing loss

**Figure 1:**
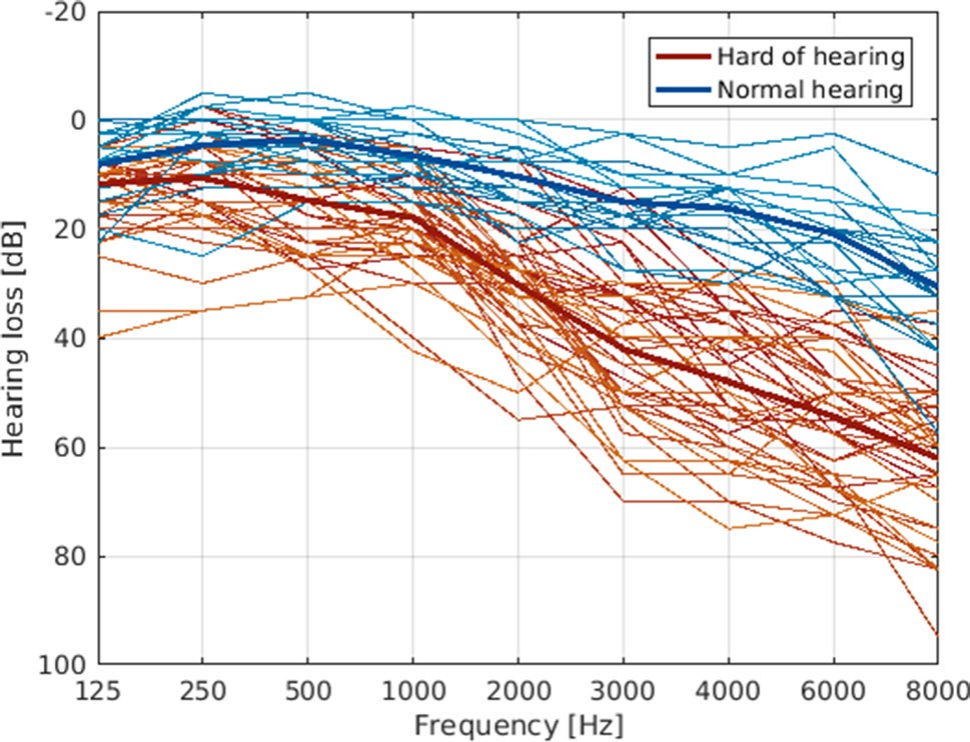
Audiograms for all subjects averaged over both ears. Normal-hearing participants are displayed in blue, hard-of-hearing participants in red with the respective group mean in bold.

Mean hearing thresholds for high frequencies (2000–8000 Hz) were 38.81 dB HL (*SD* = 15.83) when the entire sample was taken into account. The sample comprised 19 normal-hearing participants and 46 hard-of-hearing participants. Within the latter subgroup, twelve subjects exhibited hearing thresholds < 40 dB HL denoting a mild hearing loss, 26 subjects were considered to be moderately impaired (41–60 dB HL), and the remaining eight participants exhibited a severe form of age-related hearing loss (up to 80 dB HL) with a maximum hearing loss of 69.5 dB HL. Individual hearing curves for all participants can be found in Fig. 1. Hearing loss significantly correlated with age (*r* = 0.508, *p* < 0.001).

#### McGurk illusions as a function of hearing loss

**Figure 2:**
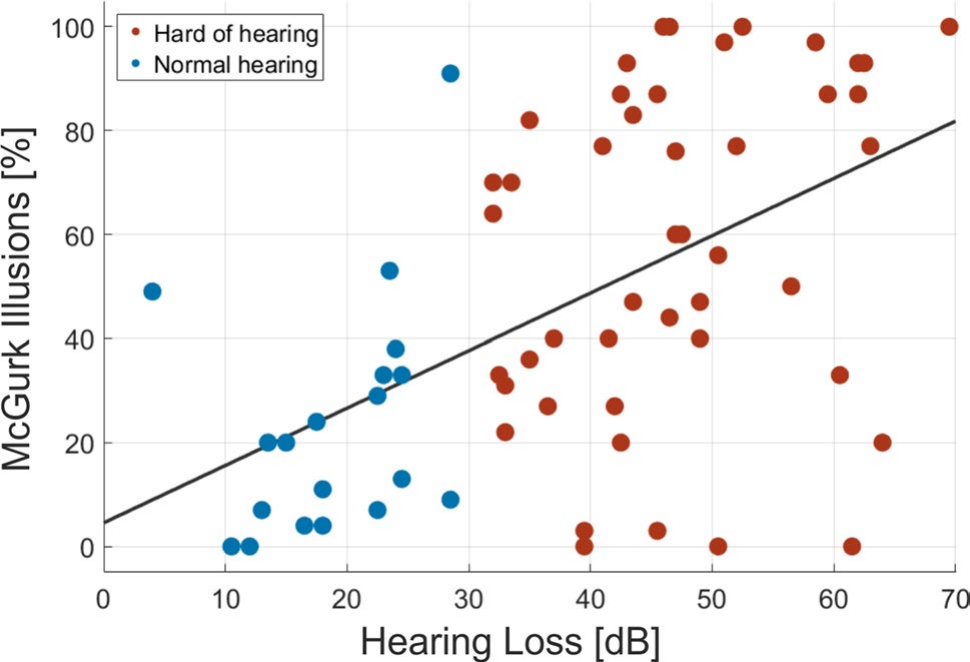
Correlation between hearing thresholds and McGurk illusion rates. For illustration, normal-hearing participants are displayed in blue, hard-of-hearing participants in red.

In response to incongruent syllable pairs, the illusionary phoneme was perceived, on average, in 47.40% (*SD* = 33.02) of all incongruent trials. McGurk illusion rates correlated positively with high frequency hearing loss (*r* = 0.522, *p* < 0.001; see Fig. 2). This effect persisted when controlling for age effects (*r* = 0.412, *p* < 0.001). In congruent trials, the correct syllable was perceived, on average, in 90.30% (*SD* = 19.53%) of the trials. Performance rates for auditory-only and visual-only conditions amounted to 87.01% (*SD* = 19.33%) and 56.99% (*SD* = 21.42%), respectively.

### Neuroimaging data

#### The effect of hearing loss on functional connectivity

**Figure 3:**
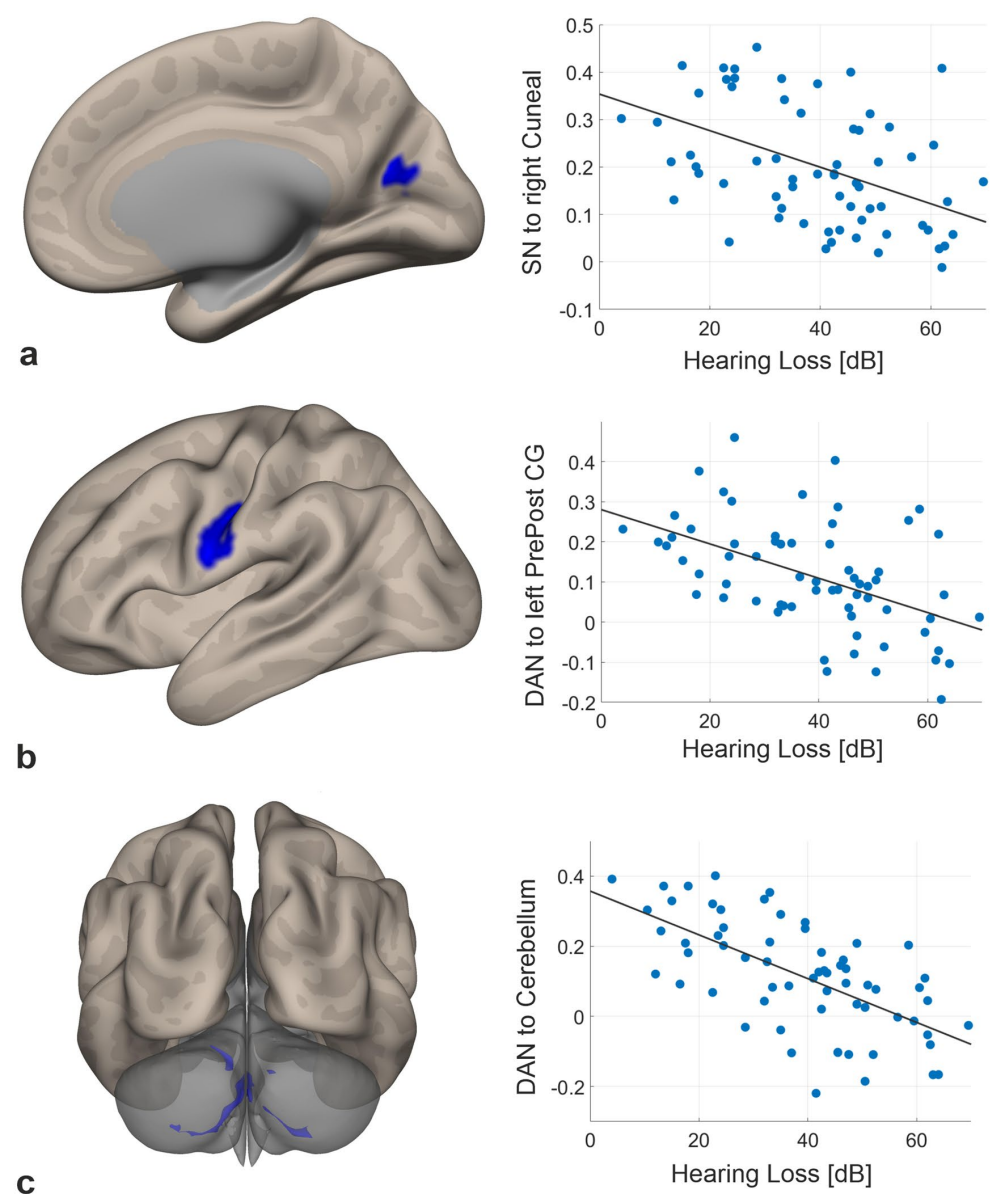
Relationship between functional connectivity and hearing loss, controlled for age. Negative correlation between hearing thresholds and resting state functional connectivity of (**a**) the salience network to the right cuneal cortex, (**b**) the DAN to a cluster in the left pre- and postcentral gyrus and (**c**) the DAN to the cerebellum. On the left, inflated brain views with the corresponding cluster activation are shown. On the right, age-corrected partial correlation plots between hearing loss and functional connectivity are displayed. *SN* = Salience Network, PrePost CG = Pre- and postcentral gyrus.

Modulatory effects of hearing loss on resting state functional connectivity was investigated considering the DAN, DMN, and salience network as well as the auditory cortices as seed regions. To control for age effects, all analyses were performed with age as covariate. Multiple regression analyses of seed-to-voxel connectivity revealed significant results for two of the assessed seeds. Salience network connectivity to a cluster covering parts of the right cuneal, intracalcarine and supracalcarine cortex negatively correlated with hearing loss (*r* = −0.46, *p* < 0.001) (Fig. 3a). Furthermore, the dorsal attention network exhibited reduced functional coupling to the cerebellum and the left pre- and postcentral gyrus with increasing hearing loss (see Fig. 3b,c). Correlation coefficients were *r* = − 0.64 (*p* < 0.001) and *r* = − 0.50 (*p* < 0.001), respectively. No significant associations were found regarding the DMN and the auditory cortices, hence no significant seed-to-voxel correlations were obtained with the seeds in the DMN and the auditory cortices. All findings including the clusters’ peak MNI coordinates, cluster and effect sizes are summarized in Table 2.

**Table 2:** Resulting clusters of multiple regression with hearing loss.

Seed region	Peak coordinates (x, y, z)	Z-score	Cluster size	Brain region
Dorsal attention network	(18, − 68, − 52)	4.34	470	Cerebellum (right)
	(− 46, − 10, 28)	4.60	327	Pre- and postcentral gyrus (left)
Salience network	(20, − 66, 18)	4.49	204	Cuneal cortex/Intracalcarine cortex (right)

#### The effect of audio-visual integration abilities on functional connectivity

**Figure 4:**
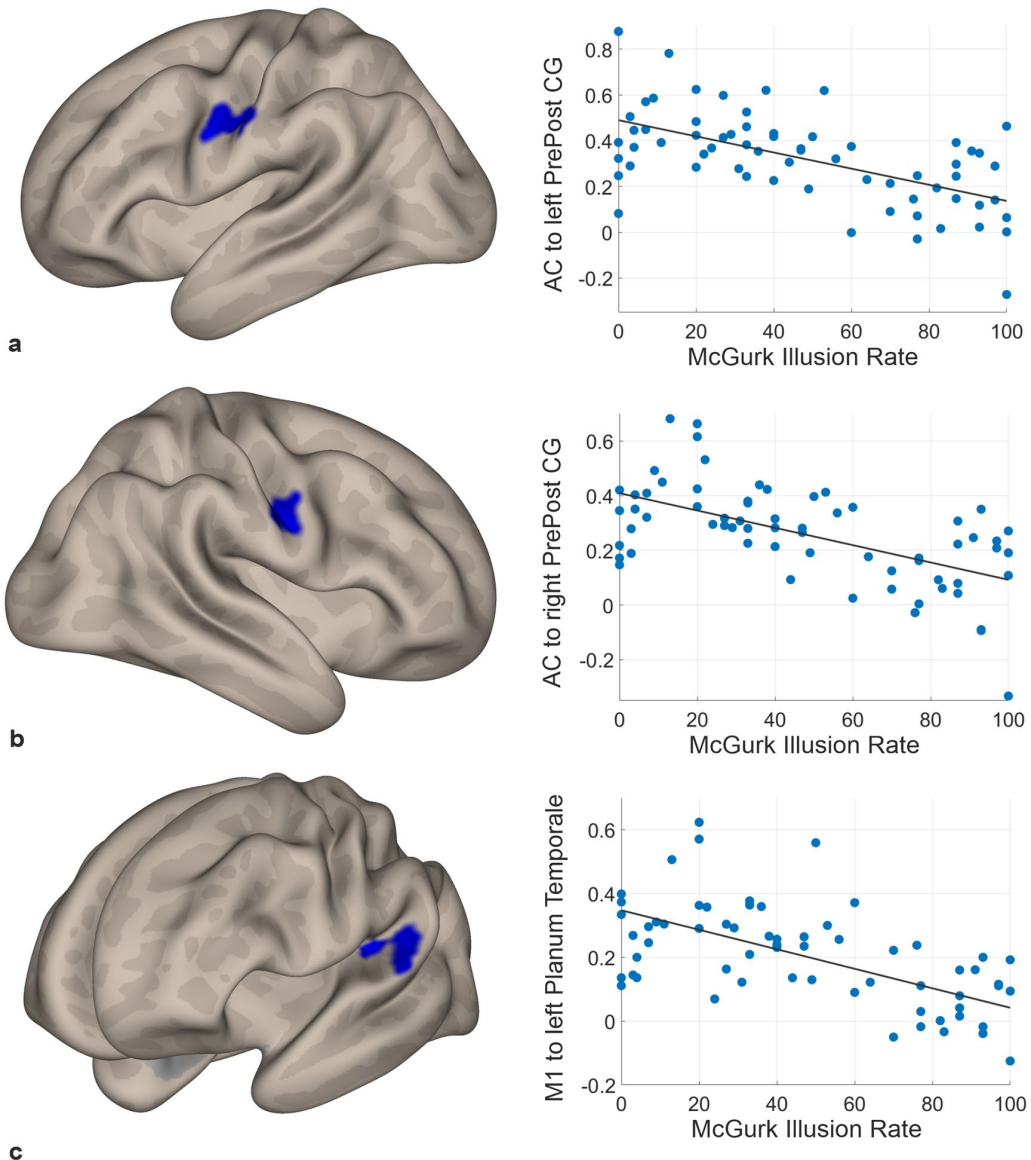
Relationship between functional connectivity and McGurk illusions, controlled for age. Negative correlation between McGurk susceptibility and functional connectivity of the auditory cortices to the (**a**) left and (**b**) right pre- and postcentral gyrus as well as the (**c**) lip area of the left primary motor cortex to the left planum temporale. Cluster activation on inflated brain views are displayed on the left, partial correlation plots between McGurk illusions and functional connectivity, controlled for age on the right side. AC = Auditory cortices, PrePost CG = Pre- and postcentral gyri, M1 = Primary motor cortex.

Functional brain connectivity as a function of McGurk illusion rates (controlling for age-effects) was investigated for three brain regions that were consistently linked to audio-visual integration (auditory cortices, lip area of the primary motor cortex, and STS). Higher McGurk susceptibility was associated with reduced coupling of auditory cortex to the left and the right pre- and postcentral gyrus (Fig. 4a,b). Correlation coefficients amounted r = − 0.58 (*p* < 0.001) and r = − 0.59 (*p* < 0.001), respectively. A similar relationship was ascertained for the seed situated in the lip area of the left primary motor cortex; higher McGurk susceptibility was related to reductions of functional coupling between the seed area in the primary motor cortex and a cluster comprising the left planum temporale and the left posterior superior temporal gyrus (r = − 0.60, *p* < 0.001) (Fig. 4c). Details of the resulting clusters are listed in Table 3. No significant results were found for the seed-to-voxel analysis of the STS.

**Table 3:** Resulting clusters of multiple regression with McGurk illusion rates.

Seed region	Peak coordinates (x, y, z)	Z− score	Cluster size	Brain region
Auditory cortices	(− 58, − 2, 30)	4.18	294	Pre- and postcentral gyrus (left)
	(50, − 6, 26)	4.45	242	Pre- and postcentral gyrus (right)
Primary motor cortex lip area	(− 54, − 38, 18)	4.56	246	Planum temporale (left)

## Discussion

The current study examined resting state functional connectivity with respect to age-related hearing loss and audio-visual integration abilities in a large sample of older adults. The two behavioural measures were positively correlated, irrespectively of any age effects. Hearing loss was found to have a negative modulatory effect on resting state functional coupling of the DAN to a cluster comprising parts of sensorimotor and primary motor cortices. The same relationship was obtained for the salience network and its connectivity to the visual cortex. Likewise, resting state functional connectivity between motor and auditory regions was negatively related to the strength of the McGurk effect.

### Reduced resting state functional coupling with progressing hearing loss

Functional connectivity at rest was influenced by age-related hearing loss. Higher hearing loss was associated with decreased functional coupling between the salience network and visual cortex and between the DAN and sensorimotor and motor regions (controlled for age effects).

Altered within-network connectivity of the salience network has been related to ageing and cognitive decline^19^, further reduced functional coupling of the salience network was also reported in fronto-temporal dementia and Alzheimer’s disease^49,50^. Our findings suggest that part of these changes may also be due to decreased hearing abilities with age. Cognitive decline associated with age-related hearing loss^5,6,8^ may, therefore, rest upon similar neural mechanisms of malfunctioning connectivity within resting state networks, as described in several neurodegenerative disorders^51^. The connectivity reductions involve the visual cortex, an area assumed to be increasingly important for hard-of-hearing elderly as they rely more strongly on visual cues^52^. The obtained reduction of coupling strength between salience network and cuneal cortex with progressing hearing loss in absence of any task might be—next to the assumed general dysfunction within the salience network—understood as a task-dependent downregulation. It is conceivable that tasks that require the detection of salient events increasingly involve the visual cortex in hard-of-hearing participants, which might be mirrored in a potential task-related increased synchronization between low-frequency fluctuations in nodes of the salience network and the visual cortex. At rest, however, when no alertness for the detection of salient events is needed, we assume that connectivity patterns reverse into anticorrelations as evident from the present analysis.

For the DAN, reductions in functional resting state connectivity in participants with hearing impairment has been shown before. Luan et al.^20^ and Husain et al.^16^ reported between-group differences in resting state connectivity of the DAN for middle-aged participants. This is the first analysis, however, indicating that elderly individuals with presbycusis exhibit progressively decreased DAN connectivity with increasing hearing thresholds. Impaired DAN connectivity might explain attentional deficits that were described in hard-of-hearing individuals^53^. Aside from that, the resulting cluster in the pre- and postcentral gyri is closely situated to brain areas for tongue, mouth and lip representations^54,55^. Further, left sensorimotor and motor cortices are part of a distributed network for speech comprehension^56^. Thus, our results suggest that functional connectivity between the DAN and areas associated with speech comprehension and production is diminished in age-related hearing loss. This may imply that the attentional focus on lip and mouth movements is altered in hearing loss.

Interestingly, in our previous investigation^13^, no effects of hearing loss on resting state functional connectivity were obtained while listening effort was found to modulate brain connectivity. This relationship was similar in normal-hearing as well as hard-of-hearing participants. Considering both studies in combination, they provide evidence for decreasing DAN connectivity with increasing hearing loss or listening effort and thus imply that different measures associated with hearing impairment (subjective listening effort as well as pure-tone audiometry) seem to impact neural coupling in similar ways. Rosemann and Thiel^13^ considered a reversal between positive and negative coupling, depending on task-based vs. resting state measures to be a possible explanation for their findings. However, not all resting state analyses reported a decline of functional connectivity in connection with hearing impairment. Increased coupling during resting state in hard-of-hearing compared to normal-hearing participants was reported by Husain et al.^16^ for the DMN and by Chen et al.^14^ for the cuneus. Puschmann and Thiel^21^ provided evidence for a continuous positive relationship of high-frequency hearing loss and resting state functional connectivity of the visual area MT+. With respect to the methodology of these investigations, it could be the case that transfer effects of directly preceding task-based recordings in Puschmann and Thiel^21^ were responsible for the persisting positive correlations during rest. Generally, comparability of the mentioned studies might be limited due to differences in sample size, age and degree of hearing loss of the group of participants. Additionally, it needs to be considered that functional connectivity of resting state networks is significantly influenced by age^19,57,58^, which was largely disregarded in the statistical analysis of previous investigations.

### Reduced resting state coupling with increased McGurk susceptibility

In line with our expectation and previous investigations^28,30^, the behavioural analysis resulted in a positive age-corrected partial correlation between McGurk susceptibility and hearing loss. It can be inferred that regardless of effects by age, hearing loss is associated with a continuous increase of audio-visual integration abilities. The behavioural outcome of increasing McGurk susceptibility was found to be neurally mirrored in reduced functional coupling between the auditory cortices and bilateral pre- and postcentral gyri, covering the lip and tongue motor region^54,55^. This finding is complemented by the results of the reduced coupling of the lip area of the primary motor cortex to the left planum temporale with increasing McGurk susceptibility. The left planum temporale constitutes an area of early auditory processing^59^. The connectivity reductions reported here affect areas that are recruited during the performance of the McGurk task^41,60^. The involvement of the primary motor cortex substantiates the motor theory of speech perception based on Liberman et al.^39^ stating that areas relevant for speech production are involved in audio-visual speech processing, probably as part of the mirror neuron system^40^. Aside from the present investigation, other studies have also described altered resting state functional connectivity of the pre- and postcentral gyri in hearing-impaired individuals, however, independent of audio-visual integration abilities^13,16,20^. When using audio-visual stimuli in task-based fMRI recordings, hearing loss was found to be positively correlated to functional coupling between auditory and visual areas when processing matching audio-visual input with respect to motion and tone pitch^21^. Further, higher McGurk susceptibility was associated with increased activity in the right precentral gyrus as well as in stronger auditory cortex connectivity in hard-of-hearing compared to normal-hearing participants for incongruent McGurk trials^29^. The current findings of bilateral auditory cortices demonstrating connectivity reductions to both left and right-hemispheric pre- and postcentral gyri cover similar areas and thus support the relevance of these brain regions for enhanced multisensory processing in presbycusis. Considering the differences in the experimental design between the mentioned investigations^21,29^ and the current study, the increased neural activity and stronger connectivity of the involved areas during the integration of audio-visual stimuli seems to be reversed into decreased functional connectivity at rest, if no integration processes take place. This may constitute a compensatory mechanism for the enhanced co-activation during task performance.

It should be noted that previous research has shown rather large variability in the McGurk illusion susceptibility, for instance based on differences in age, gender, culture or native language^61^. However, studies in elderly participants have consistently shown higher illusion rates in hard of hearing compared to normal-hearing individuals^28,30^. We recently conducted another study in which a group of participants with mild to moderate age-related hearing loss (n = 16) was measured twice with a duration of 6 months between measurements. The number of perceived McGurk illusions was highly correlated across both measurement points (yet unpublished observations). Hence, it seems that the susceptibility to the McGurk illusion seems to be relatively stable in elderly hard of hearing participants, at least for a period of 6 months. Whether the susceptibility may change with progressing hearing loss within the same participants can only be answered in a longitudinal study.

Functional connectivity of the auditory cortices was not observed to change as a function of hearing thresholds. This finding is consistent with the lacking between-group differences and correlations of auditory cortices resting state functional connectivity investigated by Husain et al.^16^ and Rosemann and Thiel^13^. In contrast, some other studies found altered connectivity of auditory seed regions in groups of people with a comparable hearing impairment^14,21,62^, however, they were inconsistent regarding the direction of effect (increased vs. decreased coupling). Based on the repeatedly reported structural and task-related changes in brain structure and activation of the auditory cortex^4,10^ as well as cross-modal reorganisations of the visual and auditory domains in age-related hearing loss^12,63^, we expected these relations to also be emulated on functional connectivity during rest. Nevertheless, in our large sample covering varying degrees of hearing loss, the hearing ability itself seems not to be linked to resting state functional connectivity in the auditory cortex. Instead, our study showed that McGurk illusion rates seem to be highly related to decreased functional connectivity. Probably, not the hearing abilities per se but rather the effect of the decreased auditory abilities on behavioural outcomes—for instance audio-visual integration abilities—seem to affect resting state functional connectivity of the auditory cortex.

## Conclusion

We here show in a large sample of elderly subjects with a wide range of untreated hearing loss that the degree of impairment is linked to changes in functional connectivity of two attention-related brain networks, the DAN and the salience network. The often observed increase in audio-visual integration, as measured with the McGurk susceptibility, was accompanied by changes in functional coupling between auditory and motor regions. For both, hearing thresholds as well as McGurk illusions, negative relationships with resting state functional connectivity were obtained. Hence, age-related hearing loss seems to be accompanied by an overall decline of connectivity affecting different brain areas and networks. Our research points out that with progressing hearing loss resting state functional connectivity continuously declines, starting already at early stages of hearing loss. The consequences of these alterations in presbycusis patients and its reversibility after hearing aid fitting are still unknown, but may become a major health concern as the prevalence of hearing loss will further increase in our ageing population.
